# The Contribution to the Assignment of Defined Daily Dose (DDD) to Antineoplastic Drugs of the Italian Working Group on DDD

**DOI:** 10.1002/pds.70379

**Published:** 2026-05-04

**Authors:** Angela Boccia, Giulia Hyeraci, Anna Girardi, Rosa Gini, Gianluca Trifirò, Ylenia Ingrasciotta, Andrea Spini, Valentina Ientile, Elisabetta Poluzzi, Giampiero Mazzaglia, Ippazio Cosimo Antonazzo, Casula Manuela, Elena Tragni, Olivia Leoni, Arianna Mazzone, Michele Ercolanoni, Martina Zanforlini, Ursula Kirchmayer, Belleudi Valeria, Marco Finocchietti, Francesco Barone‐Adesi, Giuseppe Roberto

**Affiliations:** ^1^ University of Bologna Bologna Italy; ^2^ Regional Health Agency of Tuscany Florence Italy; ^3^ University of Verona Verona Italy; ^4^ University of Milano‐Bicocca Milan Italy; ^5^ Department of Environmental and Prevention Sciences University of Ferrara Ferrara Italy; ^6^ University of Milan Milan Italy; ^7^ Lombardy Region Milan Italy; ^8^ Department of Epidemiology ASL Roma 1, SSR Lazio Rome Italy; ^9^ University of Eastern Piedmont Novara Italy

**Keywords:** antineoplastic agents, ATC, DDD, defined daily dose, drug utilization, pharmacoepidemiology

## Abstract

**Purpose:**

To report the experience of the Italian Working Group on DDD (Ita‐DDD‐wg) in assigning DDDs to a list of antineoplastic agents.

**Methods:**

Active substances from the ATC L01 group with no assigned DDD and reimbursed at least once during 2021 by the Italian Healthcare Service were identified. A DDD‐assignment reporting template was developed, including the following fields: ATC fifth‐level code, active substance, route of administration, reference indication of use, reference posology, assignment criteria, DDD calculation formula, DDD value, and DDD unit of measure. Information from national and international Summaries of Product Characteristics of medicines containing the active substance of interest, along with evidence from literature and input from Ita‐DDD‐wg experts, were considered. DDD assignment criteria were based on ATC/DDD guidelines and, whenever necessary, complemented by ad hoc criteria.

**Results:**

Forty‐three ATC 5th‐level codes were evaluated, resulting in a total of 44 DDDs assigned (2 distinct DDDs were assigned respectively to topical and oral aminolaevulinic acid formulations). For 35 out of 44 DDDs, adaptations of general WHO assignment criteria or newly defined approaches were adopted, that is, calculations based on reference body surface area (BSA = 1.82 m^2^), treatment cycle duration, and average daily amounts of topical product.

**Conclusion:**

This work demonstrated that assigning DDDs to antineoplastic agents is feasible based on few additional assumptions and criteria with respect to those from ATC/DDD guidelines. In fact, based on this work, WHO decided to establish an ad hoc working group for evaluation and assignment of DDDs to antineoplastic agents.

## Key Points

- The ATC/DDD index, maintained by the WHO Collaborating Centre for Drug Statistics Methodology, is a globally endorsed tool for generating standardized drug consumption statistics. Its correct national implementation ensures reliable, comparable data for drug utilization research.
- However, not all active substances identified with an ATC 5th level codes have been assigned a DDD because of either lack of formal request from users of the system or for other methodological reasons considered an impediment with respect to the DDD assignment the Italian Working Group on DDD was established for addressing active substances that still lack a DDD value and submit the outcome of its work for inclusion into the ATC/DDD index.
- This work demonstrated that it is feasible to assign DDDs for antineoplastic agents, even though these drugs often have high inter‐individual dose variability. They acknowledged the limitations of the DDD concept for such drugs but emphasized its value for standardized drug utilization studies.
- The Italian Working Group on DDD developed a transparent reporting template for DDD assignments and encouraged its adoption to improve the reliability of the ATC/DDD system.

## Plain Language Summary

This study describes the first effort by the Italian Working Group on Defined Daily Doses (Ita‐DDD‐wg) to help improve how we track the use of cancer medicines. The group focused on medicines used to treat cancer (called antineoplastic agents) that did not yet have a Defined Daily Dose (DDD). These DDDs help health researchers compare medicine use across different countries and time periods. The team reviewed information about each medicine's recommended doses from official documents and scientific studies and used this to calculate reasonable daily dose values. They carefully recorded how each value was determined in a clear, transparent template. In total, they suggested new DDDs for 44 medicines, sometimes using special calculations to account for body size, treatment cycles, or how the medicine is applied to the body. As a result of this project, the World Health Organization set up an ad hoc working group to assess the DDDs from Ita‐DDD‐wg and address other cancer drugs for DDD assignment. This work shows that it is possible to create useful standard daily dose values for cancer drugs, despite their complex dosing.

## 1 Purpose

The Anatomical Therapeutic Chemical (ATC) classification system and the Defined Daily Dose (DDD) are widely used standards for drug utilization research [1, 2]. The ATC system allows for the classification, grouping, and retrieval of active substances based on a five‐level hierarchical structure with increasing granularity. Most, but not all, active substances identified by an ATC 5th level codes are associated with a DDD, which represents a standard unit of measurement of drug consumption with a conversion value that may correspond to an amount of active agent (e.g., atorvastatin: ATC = C10AA05/DDD = 20 mg), a defined number of dosage units (e.g., ramipril/hydrochlorothiazide: ATC = C09BA05/DDD = 1 tablet), or an amount of pharmaceutical product (e.g., physostigmine: ATC = S01EB05/DDD = 0.4 mL of eye drops) [1, 2]. The DDD provides a standardized measure of drug use that facilitates comparisons across different populations and time periods [1]. The DDD reflects an assumed average daily maintenance dose of a drug for its main indication in adults. In some cases, if substantial differences in daily dosage do exist with respect to different route of administrations or formulations, more than one DDD may be assigned to the same ATC 5th level code [1].

The ATC/DDD index is maintained and updated annually by the WHO Collaborating Center or Drug Statistics Methodology of Oslo [1], and its use is endorsed and promoted globally by the WHO.

Implementing the ATC/DDD index in countries where medicines are mainly dispensed in standardised packages involves the mapping of each medicinal product package to the correct ATC 5th level code and the subsequent calculation of the number of DDDs contained in each package. Therefore, implementing the ATC/DDD classification system across an entire dictionary of medicinal product packages is not trivial and requires effort and resources that increase proportionally with the size of the medicinal product package dictionary to be mapped. To maximize the efficiency of this process, and also reduce the risk of errors or misinterpretations of the ATC/DDD guidelines [1], the WHO recommends the implementation and maintenance of the system to be the responsibility of a single national centre staffed with specifically trained personnel. In fact, when the ATC/DDD system is properly implemented at national level, and the resulting reference dataset is shared with users, it becomes an extremely powerful and readily available tool for generating drug consumption statistics in a standardized and widely recognised format [1, 2].

However, not all active substances identified with an ATC 5th level code have been assigned a DDD because of either lack of formal request from users of the system or for other methodological reasons considered an impediment with respect to the DDD assignment (e.g., the high inter‐individual variation of the daily amount of topical products applied for topical use). Notably, the assignment of a DDD to an ATC 5th level code occurs only upon request from interested stakeholder [2] to the WHO International Working Group for Drug Statistics Methodology [2], and must be supported by evidence‐based justification for the proposed value [1, 2].

At the end of 2021, the Italian Working Group on DDD (Ita‐DDD‐wg) was established as a spontaneous joint initiative of 20 researchers from the inter‐society group of the Italian Society of Pharmacology and the Italian Association of Epidemiology (AIE), with the aim of contributing to the development of the ATC/DDD classification system by assigning missing DDD values and proposing them for inclusion into the ATC/DDD index [3].

Therefore, the aim of this paper is to report the first experience of the ITA‐DDD‐wg regarding the assignment of DDDs to a list of ATC 5th level codes within the antineoplastics and immunomodulating agents category (ATC 2th level L01).

## 2 Methods

### 2.1 Selection of the ATC Fifth Level Codes for DDD Assignment

According to the WHO Collaborating Centre for Drug Statistics Methodology, ATC classification index with DDDs, 2025. Oslo, Norway 2025 [1], several ATC groups have historically lacked DDDs, including corticosteroids and other agents for local oral treatment, local hemostatics, appetite stimulants, blood substitutes, vasoprotectives, and antineoplastic agents. Among these, the ITA‐DDD‐wg decided to prioritize antineoplastic drugs (ATC L01) due to their relevance from National Healthcare Services (NHS), epidemiological and clinical perspectives [1]. First, the Ita‐DDD‐wg focused on active agents corresponding to ≥ 1 medicinal product reimbursed by the Italian NHS in 2021, as identified through the Farmadati database [4].

### 2.2 Reference Information Used for DDD Assignment

For each selected ATC 5th level code (i.e., active ingredient) the following reference information for DDD assignment were defined: route of administration, reference indication of use, reference posology, assignment criteria. Route of administration, reference indication of use and posology were defined based on information from Summary of Product Characteristics (SmPC) of medicinal products containing the active agent of interest and approved by the Italian Medicine Agency [5], the European Medicine Agency (EMA) [6] or the Food and Drugs Administration (FDA) [5]. Notably, to define the reference indication of use, that ideally corresponds to the indication for which the drug is expected to be most frequently used, available evidence from the published literature and knowledge from the ITA‐DDD‐wg members were also used. Finally, WHO guidelines were considered for establishing assignment criteria.

### 2.3 Presentation of Results

To ensure full transparency and reproducibility of the DDD assignment process, the Ita‐DDD‐wg developed an ad hoc reporting template including the following fields: ATC fifth‐level code, active agent, route of administration, reference indication of use, reference posology, assignment criteria, DDD calculation formula, DDD value, and DDD unit of measure.

## 3 Results

A total of 43 ATC 5th level codes from L01 group were selected for assignment. As a result, the Ita‐DDD‐wg assigned a total of 44 DDD (2 distinct DDDs were assigned respectively to topical and oral aminolevulinic acid formulations, see Table 1). Nine out of 44 DDDs were assigned based on the average recommended daily dose or the average recommended daily dose per kilogram of body weight multiplied by the reference body weight, thus according to assignment criteria defined by WHO (e.g., ATC L01XK03). The remaining 35 DDDs were assigned through the application of one or more criteria that were adapted or defined *ad hoc* for application to antineoplastic agents, that is, assignment criteria based on reference body surface area, treatment cycle duration, and average daily amounts of topical product. Notably, the ATC/DDD guidelines [1] considered 70 kg as the reference body weight while there was no mention of any reference body height value of BSA calculation method. Therefore, for drugs administered based on body weight or BSA, a reference weight of 70 kg^1^ and a reference height of 170 cm^1^ were considered, and a corresponding reference BSA of 1.82 m^2^ was obtained using the Dubois formula [7]. Finally, for the ATC L01BC52, fluorouracil combinations, which corresponded to only one authorized medicinal product containing a topical solution for transdermal administration, the Ita‐DDD‐wg decided to assign the corresponding DDD considering a hypothetical approximate mean volume of the pharmaceutical product to be applied daily based on the chosen reference indication and posology.

**TABLE 1:** List of ATC codes and the corresponding DDDs assigned by the Ita‐DDD‐wg.

ATC V	ATC V description	Route of administration	Reference indication of use	Reference posology	Assignment criteria	DDD calculation	DDD	Unit of measure
L01AB02	TREOSULFAN	Parenteral	Allogeneic haematopoietic stem cell transplantation	14 g/m^2^ for 3 consecutive days	Recommended dose*reference BSA	14 g/m^2^*1.8 m^2^	25	g
L01BB04	CLADRIBINE	Parenteral	Hairy cell leukaemia	0.09 mg/kg/die (single 7‐day treatment cycle)	Recommended dose*reference weight/day of treatment cycle	0.09 mg/kg*70 kg/die	6.3	mg
L01BC08	DECITABINE	Parenteral	Acute myeloid leukaemia	20 mg/m2 for 5 consecutive days per treatment cycle of 28 days	Recommended dose*reference BSA/day of treatment cycle	[(20 mg/m^2^*1.8 m^2^)*5 days]/28 days	6	mg
L01BC52	FLUOROURACIL, COMBINATIONS	Transdermal	Hyperkeratotic actinic keratosis	application on up to 25 cm2 of skin surface daily	Assumed mean amount of pharmaceutical product	—	2	ml
L01BC59	TRIFLURIDINE, COMBINATIONS	Oral	Colorectal cancer	35 mg/m^2^/dose twice daily on Days 1 to 5 and Days 8 to 12 of each 28‐day cycle	Recommended dose*reference BSA/day of treatment cycle	[(35 mg/m^2^ *1.8 m^2^*2)*10 days]/28 days	22	mg
L01DB11	PIXANTRONE	Parenteral	Non‐Hodgkin B‐ cell Lymphomas	50 mg/m^2^ on Days 1, 8 e 15 of each 28‐day cycle	Recommended dose*reference BSA/day of treatment cycle	[(50 mg/m^2^*1.8 m^2^)*3 days]/28 days	10	mg
L01FA01	RITUXIMAB	Parenteral	Non‐Hodgkin's lymphoma	375 mg/m^2^ every 2 months	Recommended dose*reference BSA/day of treatment cycle	375 mg/m^2^*1.8 m^2^/60 days	11	mg
L01FX02	GEMTUZUMAB OZOGAMICIN	Parenteral	Acute myeloid leukaemia	3 mg/m2 on Day 1 every 4 weeks	Recommended dose*reference BSA/day of treatment cycle	3 mg/m^2^*1.8 m^2^/28 days	0, 2	mg
L01FX04	IPILIMUMAB	Parenteral	Melanoma	3 mg/kg every 3 weeks	Recommended dose*reference weight/day of treatment cycle	3 mg/kg*70 kg/21 days	10	mg
L01FX05	BRENTUXIMAB VEDOTIN	Parenteral	Hodgkin lymphoma	1.2 mg/kg on Days 1 and 15 of each 28‐day cycle	Recommended dose*reference weight/day of treatment cycle	[(1.2 mg/kg*70 kg)*(2 days)]/28 days	6	mg
L01FD02	PERTUZUMAB	Parenteral	HER2‐positive early breast cancer	420 mg every 3 weeks	Recommended dose/days of treatment cycle	420 mg/21 days	20	mg
L01FD01	TRASTUZUMAB	Parenteral	HER2 positive metastatic breast cancer	6 mg/kg every 3 weeks	Recommended dose*reference weight/day of treatment cycle	6 mg/kg*70 kg/21 days	20	mg
L01FD03	TRASTUZUMAB EMTANSINE	Parenteral	HER2‐positive early breast cancer	3.6 mg/kg every 3 weeks	Recommended dose*reference weight/day of treatment cycle	3.6 mg/kg*70 kg/21 days	12	mg
L01FD04	TRASTUZUMAB DERUXTECAN	Parenteral	HER2‐positive breast cancer	5.4 mg/kg every 3 weeks	Recommended dose*reference weight/day of treatment cycle	(5.4 mg/kg*70 kg)/21 days	18	mg
L01FA03	OBINUTUZUMAB	Parenteral	Chronic lymphocytic leukaemia	1000 mg on Day 1 of each 28‐day cycle	Recommended dose/days of treatment cycle	1000 mg/28 days	36	mg
L01FX06	DINUTUXIMAB BETA	Parenteral	High‐risk neuroblastoma	first 10 days of each course at the daily dose of 10 mg/m2. Treatment cycle = 35 days	Recommended dose*reference weight/day of treatment cycle	(10 mg/m2*1.8 m2)/35 days	5	mg
L01FF01	NIVOLUMAB	Parenteral	Melanoma	240 mg every 2 weeks	Recommended dose/days of treatment cycle	240 mg/14 days	17	mg
L01FF02	PEMBROLIZUMAB	Parenteral	Melanoma	200 mg every 3 weeks	Recommended dose/days of treatment cycle	200 mg/21 days	9, 5	mg
L01FX07	BLINATUMOMAB	Parenteral	Acute lymphoblastic leukaemia	28mcg/die on Days 1–28 of each 42‐day cycle	Recommended dose/days of treatment cycle	(28mcg*28)/42 days	19	mcg
L01FG02	RAMUCIRUMAB	Parenteral	Gastric cancer	8 mg/kg every 2 weeks	Recommended dose*reference weight/day of treatment cycle	(8 mg/kg*70 kg)/14 days	40	mg
L01FX08	ELOTUZUMAB	Parenteral	Multiple myeloma	10 mg/kg on Days 1 and 15 of each 28‐day cycle	Recommended dose*reference weight/day of treatment cycle	[(10 mg/kg*70 kg)*2]/28 days	50	mg
L01FC01	DARATUMUMAB	Parenteral	Multiple myeloma	16 mg/kg every 28 days	Recommended dose*reference weight/day of treatment cycle	(16 mg/kg*70 kg)/28 days	40	mg
L01FB01	INOTUZUMAB OZOGAMICIN	Parenteral	CD22‐positive B cell precursor acute lymphoblastic leukaemia	0.5 mg/m2 on days 1, 8 and 15 for each 28‐day cycle	Recommended dose*reference BSA/day of treatment cycle	[(0.5 mg/m2*1.8 m2)*3 days]/28 days	0, 1	mg
L01FX10	OLARATUMAB	Parenteral	Soft tissue sarcoma	15 mg/kg on Days 1 e 8 of each 21‐day cycle	Recommended dose*reference weight/day of treatment cycle	[(15 mg/kg*70 kg) X 2]/21 days	100	mg
L01FF03	DURVALUMAB	Parenteral	Non‐Small Cell Lung Cancer	1500 mg every 4 weeks	Recommended dose/days of treatment cycle	1500 mg/28 days	54	mg
L01FF04	AVELUMAB	Parenteral	Merkel cell carcinoma	800 mg every 2 weeks	Recommended dose/days of treatment cycle	800 mg/14 days	57	mg
L01FF05	ATEZOLIZUMAB	Parenteral	Urothelial carcinoma	1200 mg every 3 weeks	Recommended dose/days of treatment cycle	1200 mg/21 days	57	mg
L01FF06	CEMIPLIMAB	Parenteral	Non‐Small Cell Lung Cancer	350 mg every 3 weeks	Recommended dose/days of treatment cycle	350 mg/21 days	17	mg
L01XD04	AMINOLEVULINIC ACID	Oral	Visualisation of malignant tissue during surgery for malignant glioma	20 mg/kg	Recommended dose*reference weight	20 mg*70 kg	1, 4	g
L01XD04	AMINOLEVULINIC ACID	Topical	Actinic keratosis of mild to moderate severity	Max 6 medicated patches (1 patch = 8 mg active substance)	Recommended dose	(6*8 + 1*8)/2	28	g
L01XJ02	SONIDEGIB	Oral	Basal cell carcinoma	200 mg (1 tablet) per day	Recommended dose	—	200	mg
L01XK03	RUCAPARIB	Oral	Epithelial ovarian, fallopian tube, or primary peritoneal cancer	600 mg twice daily	Recommended dose	600 mg*2	1.2	g
L01XX02	ASPARAGINASE	Parenteral	Acute lymphoblastic leukaemia	5000 U/m^2^ every 3 days	Recommended dose*reference BSA/day of treatment cycle	5000 U/m^2^ *1.8 m^2^/3 days	3000	U
L01XX24	PEGASPARGASE	Parenteral	Acute lymphoblastic leukaemia	2000 U/m2 every 14 days	Recommended dose*reference BSA/day of treatment cycle	2000 U*1.8 m2/14 days	260	U
L01XX41	ERIBULIN	Parenteral	Breast cancer	1.23 mg/m^2^ on Days 1 e 8 of each 21‐day cycle	Recommended dose*reference BSA/day of treatment cycle	[(1.23 mg/m^2^*1.8 m^2^)*2 days]/21 days	0.2	mg
L01XJ01	VISMODEGIB	Oral	Basal cell carcinoma	150 mg die	Recommended dose	—	150	mg
L01XX44	AFLIBERCEPT	Parenteral	Metastatic colorectal cancer	4 mg/kg every 2 weeks	Recommended dose*reference weight/day of treatment cycle	(4 mg/kg*70 kg)/14 days	20	mg
L01XG02	CARFILZOMIB	Parenteral	Multiple myeloma	27 mg/m^2^ on Days 1, 2, 8, 9, 15 e 16 of each 28‐day cycle	recommended dose*reference BSA/day of treatment cycle	[(27 mg/m^2^*1.8m^2^)*6 days]/28 days	10	mg
L01XK01	OLAPARIB	Oral	Ovarian cancer	300 mg twice daily	Recommended dose	—	600	mg
L01XG03	IXAZOMIB	Oral	Multiple myeloma	4 mg on Days 1, 8 e 15 of each 28‐day cycle	Recommended dose/days of treatment cycle	(4 mg*3 days)/28 days	0.4	mg
L01XX52	VENETOCLAX	Oral	Chronic lymphocytic leukaemia	400 mg/day	Recommended dose	—	400	mg
L01XK02	NIRAPARIB	Orale	Ovarian cancer	300 mg/day	Recommended dose	—	300	mg
L01XL04	TISAGENLECLEUCEL	Parenteral	B‐cell acute lymphoblastic leukaemia	1 infusion bag day	Recommended dose	—	1	Infusion bag
L01XY01	CYTARABINA and DAUNORUBICIN	Parenteral	Acute myeloid leukaemia	44 mg/100 mg/m^2^ on days 1, 3, 5	Recommended dose*reference BSA/day of treatment cycle	44 mg*1.82 m^2^*3days/5 days = 1 vial Or 100 mg*1.82 m^2^*3days/5 days = 1 vial—1 vial contain 44 mg cytarabine/100 mg daunorubicin	1	vial

The list of ATC fifth level codes and corresponding DDD values assigned by the Ita‐DDD‐wg was shared with the WHO Collaborating Centre for Drug Statistics Methodology of Oslo, Norway in August 2023, to be considered for inclusion in the official ATC/DDD index during the subsequent meetings of WHO International Working Group for Drug Statistics Methodology [3].

## 4 Conclusions

The Ita‐DDD‐wg initiative demonstrated that assigning DDDs to antineoplastic agents is feasible with only a few additional assignment criteria and assumptions with respect to those already outlined in the ATC/DDD guidelines [1] and applied to other active agents. For some ATC 5 ft. level codes addressed in this work, the assigned DDD values are expected to represent a reasonable approximation of the actual average daily dose used for that drug (e.g., pembrolizumab, nivolumab), at least for the reference indication considered for assignment. In other cases (e.g., olaparib, niraparib), this assumption is less likely to hold due to high inter‐individual dosage variability. Nevertheless, several substances outside the antineoplastic class were already assigned a DDD despite the high inter‐individuals and/or inter‐indications variability of the recommended daily dose (e.g., insulins, valproate, retinoids, warfarin) but still such DDDs, if used appropriately, can be considered a useful tool for DUR [8, 9]. In fact, DDDs are not intended to provide an accurate estimate of the actual number of daily doses consumed in the population of interest. Rather, understanding both the limitations of the methodology and the considered data source remains fundamental for the correct application of this standard and the reliable interpretation of resulting statistics [10, 11]. Despite such limitation, we believe that having a standard like the ATC/DDD system appropriately implemented at national level—linked to the relevant dictionary of medicinal product packages and made available to researchers—is crucial for generating comparable and reproducible drug utilization statistics across countries and over time [12]. Based on these principles, the Ita‐DDD‐wg is now focusing on the assignment of DDDs to active agents in ATC S (ophthalmologicals), which includes medications such as antineovascularization agents that also exhibit high inter‐individual variability of daily dose and dosing schedule [13].

With this project, the Ita‐DDD‐wg also developed and applied a template for the transparent reporting of the reference information based on which the DDD was assigned. We strongly encourage the adoption of this reporting approach, as we believe it can support both the evaluation of the proposed DDD values at the WHO level and the correct use of the ATC/DDD standard by end users. In fact, DDDs that are far from the relevant prescribed daily dose (PDD) can introduce substantial measurement errors in drug utilization studies. Moreover, the main indication of use of an active substance may vary from one country to another as well as across different study populations. The proposed reporting template can transparently provide useful context on background information used for DDD assignment. The extent and direction of the measurement error introduced by the application of the DDD metric should be ideally assessed in real‐world practice with respect to the actual PDD and reflect appropriate utilization patterns. Although such a validation is not always feasible, the DDD remains a technical unit of measurement designed to standardize and facilitate drug utilization research, this standard implemented once centrally and distributed and used locally by multiple users. Indeed, since not all the ATC 5th level codes have been assigned a DDD, caution is needed when aggregating data according to the 4th level of the ATC classification, particularly for antineoplastic agents.

Based on the work reported here, in early 2024 the WHO decided to set up an ad hoc Working Group on DDD for Cancer Drugs to assess the DDDs from Ita‐DDD‐wg and address other antineoplastic drugs for DDD assignment. A total of 30 new DDDs for antineoplastic agents were proposed by the ad hoc Working Group on DDD for Cancer Drugs for discussion during the 56th Meeting of the WHO International Working Group for Drug Statistics Methodology, with consideration for inclusion in the 2026 ATC/DDD index [14, 15].

## Author Contributions

G.H., A.G., R.G., G.T., Y.I., A.S., V.I., E.P., G.M., I.C.A., M.C., E.T., O.L., A.M., M.E., M.Z., U.K., V.B., M.F., F.B.‐A. and G.R. conceptualized the study, curated, and analyzed the data. A.B., G.R. drafted the original manuscript. G.H., A.G., R.G., G.T., Y.I., A.S., V.I., E.P., G.M., I.C.A., M.C., E.T., O.L., A.M., M.E., MZ, U.K., V.B., M.F. and F.B.‐A. reviewed and edited the original manuscript. All the authors approved the final version of the manuscript.

## Funding

The authors have nothing to report.

## Ethics Statement

The authors have nothing to report.

## Consent

The authors have nothing to report.

## Conflicts of Interest

The authors have no relevant affiliations or financial involvement with any organization or entity with a financial interest in or financial conflict with the subject matter or materials discussed in the manuscript. This includes employment, consultancies, honoraria, stock ownership or options, expert testimony, grants or patents received or pending, or royalties. Y.I. is the CEO of the academic spin‐off *INSPIRE srl*, which has received funding for observational studies, systematic reviews, and research activities from contract research organizations (RTI Health Solutions, Pharmo Institute N.V.) and pharmaceutical companies including Chiesi Italia, Kyowa Kirin S.r.l., Daiichi Sankyo Italia S.p.A., Grunenthal, Novo Nordisk, Shionogi, Molteni Farmaceutici, and Sanofi. G.T. has participated in advisory boards and given lectures—on topics unrelated to this manuscript—sponsored by the following pharmaceutical companies over the past two years: Egualia, Biogen, GSK, Fidia, Sanofi, Eli Lilly, Novo Nordisk, Bayer, Pfizer, AlfaSigma, and Takeda. He is also the scientific coordinator of the pharmacoepidemiology team at the University of Verona and of the academic spin‐off *INSPIRE srl*, which has carried out observational studies and research activities funded by contract research organizations (RTI Health Solutions, Pharmo Institute N.V.) and pharmaceutical companies (Chiesi Italia, Kyowa Kirin S.r.l., Daiichi Sankyo Italia S.p.A., Grunenthal, Novo Nordisk, Shionogi, Molteni Farmaceutici, and Sanofi). Additionally, he served as a consultant for Viatris in a legal case related to an adverse reaction to sertraline.

## References

[1] “ATCDDD – Guidelines” 2026. [Online] https://atcddd.fhi.no/atc_ddd_index_and_guidelines/guidelines/.

[2] “ATCDDD – Home” 2025 https://atcddd.fhi.no/.

[3] “Implications of a Defined Daily Dose Fixed Database for Drug Utilization Research Studies: The Case of Statins in Portugal – PubMed” 2025 https://pubmed.ncbi.nlm.nih.gov/33576512/. 10.1111/bcp.14770 33576512

[4] Farmadati 2025 https://www.farmadati.it/.

[5] European Medicines Agency (EMA) 2023 https://www.ema.europa.eu/en/homepage.

[6] Commissioner of the. U.S. Food and Drug Administration. FDA 2025 https://www.fda.gov/.

[7] D. Du Bois and E. F. Du Bois , “A Formula to Estimate the Approximate Surface Area if Height and Weight Be Known. 1916,” Nutr Burbank Los Angel Cty Calif. 5, no. 5 (1989): 303–311. 2520314

[8] S. A. Hollingworth and M. J. Eadie , “Antiepileptic Drugs in Australia: 2002‐2007,” Pharmacoepidemiology and Drug Safety 19, no. 1 (2010): 82–89, 10.1002/pds.1871. 19802824

[9] A. M. Moura , S. O. Martins , and J. F. Raposo , “Consumption of Antidiabetic Medicines in Portugal: Results of a Temporal Data Analysis of a Thirteen‐Year Study (2005‐2017),” BMC Endocrine Disorders 21, no. 1 (2021): 30, 10.1186/s12902-021-00686-w. 33627117 PMC7905618

[10] T. Grimmsmann and W. Himmel , “Discrepancies Between Prescribed and Defined Daily Doses: A Matter of Patients or Drug Classes?,” European Journal of Clinical Pharmacology 67, no. 8 (2011): 847–854, 10.1007/s00228-011-1014-7. 21544512 PMC3134712

[11] P. Ihle , K. Krueger , I. Schubert , et al., “Comparison of Different Strategies to Measure Medication Adherence via Claims Data in Patients With Chronic Heart Failure,” Clinical Pharmacology & Therapeutics 106, no. 1 (2019): 211–218, 10.1002/cpt.1378. 30697693 PMC6617982

[12] “EMMA PROJECT: Exposure to Medications Measured Using Atc/DDD Classification System. Agenzia Regionale di Sanità Toscana” 2025 https://www.ars.toscana.it/news‐ns/5199‐il‐progetto‐emma‐esposizione‐ai‐farmaci‐misurata‐utilizzando‐il‐sistema‐di‐classificazione‐atc‐ddd.html.

[13] “Abstract Book ‐ Uppsala University” 2025 https://www.uu.se/en/department/pharmacy/research/pharmacoepidemiology/eurodurg‐2025/abstract‐book.

[14] “ATC 56th Executive Summary” 2025 https://www.who.int/publications/m/item/inn‐24‐607.

[15] ATCDDD ‐ Lists of Temporary ATC/DDDs and Alterations 2025 https://atcddd.fhi.no/lists_of__temporary_atc_ddds_and_alterations/.

